# Undersampling Taxa Will Underestimate Molecular Divergence Dates: An Example from the South American Lizard Clade Liolaemini

**DOI:** 10.1155/2013/628467

**Published:** 2013-10-09

**Authors:** James A. Schulte

**Affiliations:** Department of Biology, 8 Clarkson Avenue, Clarkson University, Potsdam, NY 13699, USA

## Abstract

Methods for estimating divergence times from molecular data have improved dramatically over the past decade, yet there are few studies examining alternative taxon sampling effects on node age estimates. Here, I investigate the effect of undersampling species diversity on node ages of the South American lizard clade Liolaemini using several alternative subsampling strategies for both time calibrations and taxa numbers. Penalized likelihood (PL) and Bayesian molecular dating analyses were conducted on a densely sampled (202 taxa) mtDNA-based phylogenetic hypothesis of Iguanidae, including 92 Liolaemini species. Using all calibrations and penalized likelihood, clades with very low taxon sampling had node age estimates younger than clades with more complete taxon sampling. The effect of Bayesian and PL methods differed when either one or two calibrations only were used with dense taxon sampling. Bayesian node ages were always older when fewer calibrations were used, whereas PL node ages were always younger. This work reinforces two important points: (1) whenever possible, authors should strongly consider adding as many taxa as possible, including numerous outgroups, prior to node age estimation to avoid considerable node age underestimation and (2) using more, critically assessed, and accurate fossil calibrations should yield improved divergence time estimates.

## 1. Introduction

Alternative taxon sampling strategies are known to affect many facets of phylogenetic reconstruction [1–3]. Linder et al. [4] conducted one of the most thorough analyses assessing the impact of taxon sampling on divergence date estimation using resampling analyses. This study found that mean estimated ages of focal nodes were significantly younger with sparser taxon sampling than when taxa from the complete dataset were included. In addition, the more distant the focal node was from the calibration point, the more sensitive estimation effects were on nodal ages if the taxa were undersampled, especially for nonparametric rate-smoothing (NPRS) methods; but penalized likelihood (PL) and Bayesian methods also were prone to these effects especially if undersampling was large. Subsequent studies following up on these important results are lacking in the literature. This is especially important because numerous multigene studies estimate divergence times but often significantly undersample large clades using a single representative species in place of higher taxonomic groups. I argue that unless these large clades are densely sampled and calibration points are carefully chosen and spread across the tree, node divergence times in most cases will be underestimated in these studies.

 The sampling scheme of Linder et al. [4] did not address two important aspects of divergence time estimation. First, the effect of undersampling different numbers within specific clades could not be rigorously tested because they analyzed six smaller subsets created by selectively deleting terminals from the complete 300 species trees. Second, the effect of number of fossil calibrations on estimated divergence times was not assessed as only a single basal node was used as a calibration point. Use of a single fossil calibration may result in greater error in lineage rate estimates if multiple calibrations are employed. Thus, there is general consensus among systematists that the more calibrations spread across the tree, the more accurate the divergence times. In addition, numerous authors have strongly emphasized the importance of critical evaluation of all fossil calibrations prior to use [5–7]. Unfortunately, for many taxa, the fossil record is very sparse and numerous fossils may not be available given taxon sampling in a particular study.

One strategy to circumvent the problem of too few fossil calibrations is to sample additional taxa outside the ingroup where additional fossils may be available. So that, even if the calibrations are a greater distance away from the focal clade, PL and Bayesian methods may be able to accommodate rate heterogeneity across a large phylogeny as long as the ingroup is not drastically undersampled. For many species, this is a feasible strategy given the large amount of molecular sequence data collected over the last 30 years and their availability in public databases such as NCBI, TreeBase, or Dryad. I employ this strategy to obtain a revised timeline of diversification within the species-rich clade of South American iguanid lizards Liolaemini.

Liolaemini is composed of three genera, *Ctenoblepharys*, *Phymaturus*, and* Liolaemus.* The latter two groups contain the bulk of diversity with approximately 37 *Phymaturus* species and at least 230 *Liolaemus* species currently recognized. They are distributed throughout the southern half of South America in almost every habitat from Peru to Tierra del Fuego. Within *Liolaemus*, two subclades (or subgenera) are recognized: (1) *Liolaemus*—with species mostly distributed west of the Andes; (2) *Eulaemus*—with species distributed mostly east of the Andes; however, both groups contain taxa that cross into low elevation areas on the opposite side or occupy higher elevations. Liolaemini lizards also exhibit broad diversity in morphological, ecological, physiological, and life history traits that make them an ideal group to address a wide variety of evolutionary questions. 

To address these questions, it is essential to have an accurate estimate of diversification times within the group. Schulte II et al. [8] were the first to use molecular sequence data to date divergence within *Liolaemus*. This study estimated the divergence time between subgenera to be at least 12.6 million years ago based on an mtDNA molecular evolutionary rate. However, this should be considered a minimum estimate with the true divergence age likely to be older. Schulte II and Moreno-Roark [9] estimated crown ages of viviparous clades in Liolaemini to be between 3 and 52 MYA for several viviparous *Liolaemus* clades and approximately 66 MYA for *Phymaturus*. In each of these two studies, at least 62 Liolaemini taxa were sampled representing almost all major lineages. A recent analysis of 17 taxa in the subgenus *Eulaemus* and two outgroups from the subgenus *Liolaemus* estimated a crown *Eulaemus* clade age of 18.08 MYA with subsequent diversification of the major species groups occurring between 2.97 and 8.1 MYA [10]. These node ages were much younger than those estimated by Schulte II and Moreno-Roark [9]. The discrepancies in divergence times estimated from these studies may be related to a number of factors, such as taxon sampling size differences (62 versus 17), number of fossil calibrations (several versus one), time estimation methods (PL versus Bayesian), or sequence data sources (mtDNA versus combined nuclear and mtDNA). For the present study, I examine the first three issues. At present, there is no consensus whether mitochondrial or nuclear DNA or a combination of both yield more accurate divergence times [11]. This issue begs further study and is beyond the scope of this study.

There are four main goals in this work. First, the effect on several clade age estimates within iguanid Liolaemini lizards is examined by undersampling taxa within individual clades by randomly subsampling within those clades. It is expected that when fewer ingroup species are sampled, node ages will be younger compared to complete sampling. Next, I examine the effect of using different numbers of fossil calibrations on those same clade ages and alternative sampling schemes. Node ages are expected to show greater variance when fewer calibrations are used and clades are drastically undersampled then with the full complement of fossil calibrations and full sampling. Third, compare results of PL and Bayesian methods when different numbers of fossil calibrations are used to estimate nodal ages. Finally, this analysis will generate a revised timing of diversification of most major clades within Liolaemini that can be used by future researchers interested in exploring the history of speciation, biogeography, and evolution of this diverse clade. This study uses a phylogeny of 209 previously published squamate reptile mtDNA sequences, primarily within family Iguanidae, to reconstruct divergence times and address these four goals. 

## 2. Methods

### 2.1. Taxon and Molecular Sampling

Mitochondrial DNA sequences representing 92 Liolaemini taxa (here considered the ingroup) including almost all major lineages, as well as 110 outgroup species from all other clades within Iguanidae, three representatives from Acrodonta, and four additional outgroups outside Iguania, are used in phylogeny and divergence time estimation. GenBank accession numbers for all species are presented in Supplemental Material (see the Supplementary Materials at http://dx.doi.org/10.1155/2013/628467). Specimen voucher, locality information and citation information are available from GenBank flat files.

These sequences represent the mitochondrial-encoded region spanning ND1 to COI. For this analysis, the protein-coding regions, part of ND1, all of ND2, and part of COI were used, as well as the seven intervening tRNA regions. DNA sequences were aligned manually and then translated to amino acids using Mesquite v. 2.75 [12] for confirmation of alignment and to check for premature stop codons. tRNA secondary structure models were used to assess alignment of all tRNAs. Aligned sequence base positions inferred to have ambiguous homology at the ends of ND1, ND2, and several loop regions in tRNAs were excluded from phylogenetic analyses (263 out of 1862 aligned positions—14%) for a final dataset of 1599 aligned base pairs. Alignment is available in TreeBASE (http://purl.org/phylo/treebase/phylows/study/TB2:S14692).

### 2.2. Phylogenetic Analyses

Phylogenetic trees were estimated using maximum likelihood (ML) with RAxML [13] and MrBayes 3.2.1 in the CIPRES portal [14]. PartitionFinder [15] was used to determine the best-fit model of molecular evolution and partitioning scheme with four independent runs. This program selects the best-fit partitioning scheme and DNA sequence models based on predefined data blocks and using alterative information criteria. This program combines the tedious steps of performing ModelTest runs and comparisons of different a priori partitions into one analysis. *A priori* partitions compared in PartitionFinder were each codon position (1st, 2nd, and 3rd) for each protein coding gene (ND1, ND2, and COI) plus one partition for all tRNA positions (10 partitions total). Model parameter values were estimated from the data. Bootstrap resampling was applied using RAxML with 100 pseudoreplicates and parameter values estimated for each pseudoreplicate. Search conditions were identical to the initial search. The detailed bootstrap tree is available in supplementary material. We considered a bootstrap value of 95% as strongly supported [16], <95 to 70% as moderately supported, and <70% as weakly supported. 

Bayesian phylogenetic analyses were performed in MrBayes 3.2.1 [17] using the same sequence evolution model and partitioning scheme as the ML analyses. Four independent runs of 20 million generations and four Markov chains with default heating values were used. Parameter values for the model were estimated from the data and most were initiated with default uniform priors except that branch lengths were unconstrained (no molecular clock) with default exponential priors. Trees and parameter values were sampled every 1000 generations resulting in 20000 saved trees per analysis, of which the first 25% were discarded as “burn-in”. Stationarity was assessed by plotting the −ln⁡⁡*L* per generation in the program Tracer 1.5 [18] and using the average standard deviation of split frequencies implemented in MrBayes. If the four runs are converging on the same tree, the average standard deviation of split frequencies is expected to approach zero. After confirming that the analysis appeared to reach stationarity, the 60000 trees (15000 from each run after burn-in) were used to calculate Bayesian credibility values (BC) for each branch in a 50% majority-rule consensus tree. Clades with BC ≥95% were considered strongly supported with the caveat that BC may overestimate support for reasons discussed in [19–21].

### 2.3. Divergence Time Estimation

If significant rate variation is estimated on branches throughout the phylogenetic tree, relaxed clock methods are appropriate. To test whether relaxed clock models are a significantly better fit than a strict clock model, MrBayes was used to generate the posterior distribution of trees with and without enforcing a strict clock. Analytical parameters were similar to those detailed above, and log_10_ Bayes factors (BFs) calculated in Tracer were used to compare the two sets of trees. Because there is significant rate variation across the tree (see Bayesian results below), two methods were used to estimate divergence times. 

First, Penalized Likelihood (PL) implemented in r8s version 1.8 [22] was used. PL has been shown to be robust to modest model violations, is easily implemented in a single software package, and performs as well or better than other rate heterogeneity methods such as Bayesian and nonparametric rate smoothing in numerous empirical and simulated data sets [4, 7, 23–26]. The overall highest maximum likelihood tree was used as a fixed, backbone tree to obtain point divergence time estimates as well as the backbone tree for bootstrapped node age estimates (see below). Crossvalidation analyses to determine the optimal smoothing parameter as outlined by Sanderson [23] were computationally impractical. However, a previous study [9] found a smoothing parameter value of 0.9 to be optimal using similar taxon and nucleotide data sampling. I also tested a range of smoothing parameters from 0.5 to 2 and found that node age estimates differed by less than one million years for all nodes in the focal group (Liolaemini) across that range of smoothing parameter values. All r8s analyses implemented the logarithmic penalty function and truncated Newton method. Ten internal nodes were assigned minimum age estimates corresponding to fossil calibrations determined primarily by whether a consensus exists among paleontologists on the relative position of taxa in the squamate phylogeny. Minimum age estimates were assigned to stem groups, the most inclusive group of taxa that contains all extant and extinct clade members [27]. PL requires at least one node that is either fixed or set to a maximum age, so the node age of Iguania was assigned a maximum age of 218 MYA and minimum age of 144 MYA. These dates were chosen to span the age of Iguania inferred from several previous studies dating this node's age using mtDNA, nuclear DNA, or both. All other calibrations were set as minimum ages (see the appendix for dates, fossils, and supporting references). Divergence time confidence intervals on the highest likelihood tree were assessed using the bootstrap (100 replicates) method outlined in Sanderson [22] using PAUP* [28] and r8s.

The second method implemented Bayesian node age estimation using MrBayes 3.2.1 to compare with results from PL estimates. The same eleven calibrations used in PL analyses were implemented for Bayesian analyses. A uniform probability distribution on the age of each calibration node was used with the minimum set to the age of the oldest fossil that could be confidently assigned to the stem age of that clade and a maximum age of 218 million years, which is the maximum age of Iguania inferred from previous studies (see above). This calibration age probability distribution is preferred here because the only information that can be incorporated into node dating analysis with confidence is the minimum stem age of the fossil. A fixed distribution was not used because fossils only give information on the minimum age of a clade [5, 6] and never its actual age of origin. An offset exponential distribution was not used because although the minimum age information is incorporated with this parameter, the rate component cannot be confidently assigned a value. Therefore, we prefer to use the “uninformative” prior of a uniform distribution age prior for all 11 calibration points. 

We explored three relaxed clock models implemented in MrBayes 3.2.1 to determine which was the best fit for the combination of these data and calibration points. The models are the Thorne-Kishino 2002 (TK02) model [29], compound Poisson process (CPP) model [30], and the independent gamma rates (IGR) model [31]. The TK02 model is a continuous autocorrelated model similar to the one implemented in multidivtime [29, 32]. The CPP model is a discrete autocorrelated model similar to the model implemented in PhyloBayes [33]. The IGR model is a continuous uncorrelated model where branch rates are drawn independently from a gamma distribution [18, 31]. This model is similar (though not identical) to the model implemented in BEAST. Therefore, none of these mentioned software packages were used as the relaxed clock models implemented by them could be evaluated with the following MrBayes analyses. 

To identify which relaxed clock model was the best fit for this dataset, and 11 calibration points, I conducted analyses as in the nonclock analyses above using four independent runs of four parallel chains each for each of the three models using all calibration points. Burn-in was set to 25% of samples and discarded prior to comparisons of the distribution of posterior samples using Bayes factors calculated in Tracer. Preliminary analyses showed the CPP model to be a poorer fit than either the TK02 and IGR models under a variety of conditions and is not considered further. Two additional parameters implemented in the TK02 and IGR models were examined to determine whether there was a better fit to the data. These were the TK02varpr and IGRvarpr. These parameters estimate the rate at which variance of the effective branch length increases over time under the respective models. For such a large dataset, it is be possible that large rate differences in effective branch lengths across the tree could affect divergence time estimates in different parts of the tree. For both TK02varpr and IGRvarpr parameters, the exponential and uniform distributions were evaluated using the default priors of 10 and (0,1), respectively, for each of the distributions.

### 2.4. Alternative Taxon and Calibration Subsampling Schemes

To examine the effect of undersampling taxa on divergence time estimates within Liolaemini, three resampling sets were produced using the APE package version 3.0–7 in R [34]. For each of the three sets, different numbers of taxa were randomly deleted from only the large *Liolaemus* clade in which 85 taxa were sampled here, but all other ingroup and outgroup taxa were included as these are where most of the calibration points lie. The three sets of sampling schemes were as follows: (1) for the 85 taxon clade corresponding to genus *Liolaemus*, 3, 5, 15, 30, 50, 70, 80, and 82 taxa were randomly deleted from the clade; (2) for the clade corresponding to subgenus *Eulaemus* (49 species), 3, 5, 15, 30, 44 and 46 taxa were randomly deleted; and (3) for the clade corresponding to subgenus *Liolaemus* (36 species), 3, 5, 15, 30, and 33 taxa were randomly deleted. All subsampled datasets had to be manually manipulated to obtain summaries of results, so 25 replicates per sampling scheme (taxon deletion set) were produced. This number is expected to yield relatively accurate mean and standard deviation estimates of nodes ages on subsampled datasets. For each taxon deletion set, a file was created that contained the aligned sequence of the sampled taxa and the associated tree without the deleted taxa. These files were then analyzed in PAUP* using ML (without a priori partitions due to computational limitations) and the GTR+Γ+I model to obtain estimates of branch lengths on each of the subsampled trees. All trees were analyzed in r8s using the same conditions as with the full tree to obtain mean and standard deviation divergence time estimates for each subsampling scheme using the PROFILE command in r8s [35]. *Mabuya*, *Cnemidophorus*, *Elgaria*, and* Varanus* outgroup sequences were removed prior to PL analyses to prevent overestimation of the evolutionary rate across the phylogeny. For this part of the study, only PL in r8s was used to assess the affect of undersampling taxa on divergence time estimates as analyzing all the resampled datasets with the current implementation of MrBayes and manipulation of results files was computationally impractical.

Another goal of this study is to examine the effect of using fewer calibrated nodes on divergence time estimates. For this part of the study, we used two alternative strategies. First, we reran all three sets of subsampled trees in r8s using only the node representing the common ancestor of Iguania set with a maximum age of 218 MYA and minimum age of 144 MYA and all other nodes without calibrations. The second strategy used only two calibrations, the root node calibrated as above and the *Pristidactylus* fossil described by Albino [36] from the Early-Mid Miocene set at a minimum age of 16 MYA (see the appendix). This fossil was chosen rather than the *Liolaemus* fossil from the same horizon because of the remaining nine sampled fossil calibrations; it is the phylogenetically closest group, and the subsampled datasets made the use of the *Liolaemus* fossil impractical computationally. Again only PL using r8s was used to analyze all three alternative subsampling sets under the different numbers of calibrations used. However, MrBayes was used to assess the affect of different numbers of calibrations on divergence time estimates for the complete dataset. That is, MrBayes analyses performed as above using the optimal molecular clock (see below) were run using the complete set of 11 calibration points, only the Iguanian node calibration, and the Iguania plus *Pristidactylus* fossil calibration. 

## 3. Results

### 3.1. Phylogenetic Estimation

**Figure 1:**
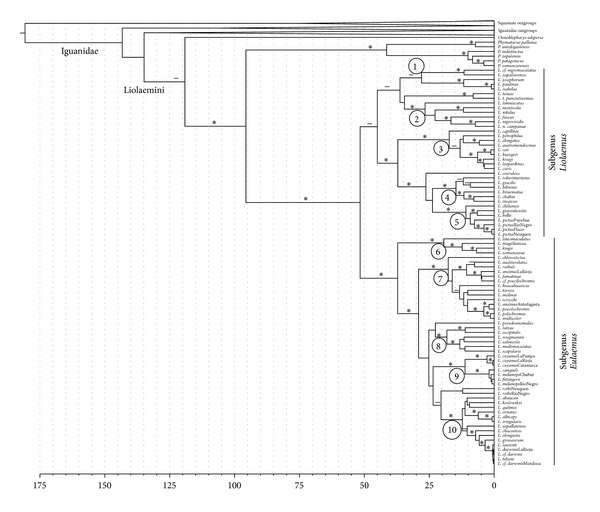
Phylogenetic relationships and timetree for taxa within the iguanid lizard clade Liolaemini. A single ML topology (−ln⁡⁡*L* = 98478.29) was obtained from analysis of 1599 aligned base positions of ND1-COI data (1283 distinct site patterns) using the best-fit partitioning scheme found with PartitionFinder and GTR+Γ+I for all partitions. Asterisks above branches denote bootstrap values between 95 and 100%, dashes above branches denote bootstrap values between 94 and 70%, and branches without notation have bootstrap values below 70%. This timetree represents the point estimate of node divergences among Liolaemini taxa and does not include mean and standard deviation values from bootstrapped trees (see Tables 1–5). Branches labeled with circled numbers represent the following major clades: (1) *L. nigromaculatus* group, (2) *L. lemniscatus* group, (3) *L. elongatus-L. kriegii* complexes, (4) *L. bibronii-L. alticolor* groups, (5) *L. chiliensis* group, (6) *L. lineomaculatus* section, (7) *L. montanus* series, (8) *L. wiegmannii* series, (9) *L. melanops* group, and (10) *L. darwinii* complex.

The best-fit partitioning scheme using the Akaike Information Criterion in PartitionFinder had a value of −ln⁡⁡*L* = 98586.62 and found six partitions and a GTR+Γ+I model (except partitions 1 and 6 which found GTR+Γ to be optimal) best explained the data: (1) third codon positions in ND1 and ND2; (2) first codon position ND2; (3) second codon position ND2; (4) tRNA positions; (5) first codon positions in ND1 and COI, third codon positions in COI; (6) second codon positions in ND1 and COI. However, subsequent ML and Bayesian analyses assumed a general time reversible sequence evolution model with gamma distributed rate variation and proportion of invariant sites (GTR+Γ+I) for all partitions. A single topology was found (−ln⁡⁡*L* = 98478.29, Figure 1) in the RAxML analysis of ND1-COI data using this partitioning scheme with GTR+Γ+I for all sites. 

Relationships among the major groups of Liolamini lizards are generally consistent with previous hypotheses recovered using this region of mtDNA [8, 9, 37]. Monophyly of *Phymaturus* and *Liolaemus* (100% bootstrap) as well as their sister taxon relationship (99% bootstrap) are well supported. There is weak bootstrap support (76%) for the monotypic genus *Ctenoblepharys* as the sister taxon to the clade containing *Phymaturus* and *Liolaemus*. Within *Liolaemus*, the monophyly of the two subgenera, *Liolaemus* and *Eulaemus*, are each recovered with moderate and strong support (94% and 100% bootstrap, resp.). 

Five primary clades are highlighted in the subgenus *Liolaemus *(Figure 1). The first group is moderately supported (93%) and contains taxa generally distributed in Northern Chile and includes *L. cf. nigromaculatus*, *L. zapallarensis*, *L. josephorum*, *L. paulinae*, and *L. isabelae*. The sister group to this clade contains both *L. tenuis *and* L. t. punctatissimus* and a well supported (98% bootstrap) clade containing *L. leminscatus*, *L. monticola*, *L. nitidus*, *L. fuscus*, and* L. nigroviridis.* All of the latter species are mostly distributed at a wide range of elevations in central Chile. The third well-supported group is composed of taxa distributed primarily at mid and high elevations in the central and southern Andes and includes *L. capillitas*, species in the *L. elongatus* complex, and *L. ceii *and* kriegi* species complexes. The sister group to the latter clade is composed of *L. coeruleus* as the sister taxon to two well-supported groups. A monophyletic group comprising a diverse group of small bodied taxa that occupy the highest elevations in the Andes and low elevations in southern Argentina includes species from the *L. alticolor* and *bibronii* species groups. The final clade is weakly supported (61% bootstrap) as the sister group to the previous well-supported (100%) clade and contains taxa that occupy scrub and forest habitats in central and southern Chile, such as *L. chiliensis*, *L. gravenhorstii*, *L. bellii*, and *L. pictus*.

Subgenus *Eulaemus* is composed of taxa primarily in the Andes and eastern lowlands with five principal clades emphasized here (Figure 1). A monophyletic group containing species from generally high latitudes in the eastern lowlands corresponding to the *L. lineomaculatus* section (*L. lineomaculatus*, *L. magellanicus*, *L. kingii*, *and L. somuncurae*) of Schulte II et al. [8] is strongly supported as monophyletic and the sister group to the remaining *Eulaemus* species (100% bootstrap). Among the remaining species is a large well-supported monophyletic group corresponding to the *L. montanus* series [8] and includes taxa distributed primarily at mid- to high elevations on the Puna Plateau. The remaining species in the eastern clade are contained mostly in three well-supported groups (100% bootstrap), the *wiegmannii* series, a monophyletic group corresponding to the *melanops* series of Fontanella et al. [10], and a clade containing species in the *L. darwinii* species complex. *Liolaemus pseudoanomalus* and two representatives of the *L. rothi* species group also are recovered in the remaining species with *Eulaemus*, but their phylogenetic position is not well supported in this analysis.

The harmonic mean −ln⁡⁡*L* of the consensus topology of the 60000 trees from the posterior distributions of nonclock analyses using MrBayes was 102558.23. The topology of the ingroup (Liolaemini) was generally very similar to the nonclock ML analyses with a few minor exceptions. Those exceptions were alternative positions in the two topologies of the following clades or single taxa: (1) *Liolaemus tenuis *and* L. t. punctatissimus*; (2) *L. pseudoanomalus*; and (3) relationships of *L. abaucan*, *L. koslowskyi*, and* L. quilmes *with the* L. boulengeri* series. In addition, all differences between the nonclock MrBayes consensus topology and the ML topology were poorly supported with ML bootstraps and Bayesian credibility (BC) values below 95%.

### 3.2. Divergence Time Results Using All Fossil Calibrations

The comparison of the posterior distribution of trees from Bayesian analyses with and without a strict molecular clock enforced found that the distribution of trees generated from enforcing a strict molecular clock was significantly worse fit than without a strict clock enforced (log_10_BF values between 99–101). Therefore, it is appropriate to use relaxed clock models with this dataset. 

Penalized Likelihood (PL) divergence time analyses using the overall highest ML tree and bootstrapped branch lengths and all calibrations revealed that the mean divergence time for crown clade Liolaemini was 111.3 MYA (SD = 10.8), which is good within the Early Cretaceous Period. Within Liolaemini, crown *Phymaturus* and *Liolaemus* diverged 39.6 MYA (SD = 5.4) and 47.6 MYA (SD = 5.9), respectively. The divergence time estimates for the initial splits within the crown subgenera *Liolaemus* (38.9 MYA (SD = 5.6)) and *Eulaemus* (35.8 MYA (SD = 4.8)) occurred in the late Eocene. For the 10 primary clades that comprise the majority of diversity within genus *Liolaemus*, divergences occurred almost entirely in the Miocene with the earliest divergence at the Oligocene-Miocene boundary (24.0–9.9 MYA). This range of node ages is contained entirely within the five clades of subgenus *Liolaemus* with the *L. nigromaculatus* group species diverging earliest at 24 MYA (SD = 3.9) and the *L. chiliensis* group species diverging in the early Late Miocene 9.9 MYA (SD = 2.3). Within the subgenus *Eulaemus*, the earliest divergence was the *L. lineomaculatus* series (18.8 MYA (SD = 3.2)) in the Early Miocene and the remaining splits occurring in the Early to Middle Miocene 17.1 to 11.5 MYA.

The IGR relaxed clock model provided the best fit of these data and calibration points over the CPP and TK02 using log_10_BF calculated in Tracer (Results not shown—available upon request from the author). Within the IGR model, log_10_BF did not strongly favor either the exponential or uniform distribution priors for the branch length rate variance parameter. Thus, both rate variance parameters were run for the IGR molecular clock model and their results on molecular divergence time estimates are presented in Table 6.

When all 11 calibrations and either the exponential or uniform IGRvarpr was used, in all cases, divergence time estimates for the five focal nodes within Liolaemini were older than PL estimates. The greatest age estimate difference between the IGR-exponential model and PL was for the subgenus *Liolaemus*. Comparing the IGR-exponential model to the IGR-uniform model, in all cases, the uniform model yielded age estimates younger than those generated from the exponential model. However, it should be noted that given the error typically associated with divergence time estimation using any method, the standard deviations of the bootstrapped PL estimates strongly overlap with all 95% posterior credibility intervals from Bayesian analyses.

### 3.3. Effect of Taxon Sampling on Divergence Time Estimates

Substantial differences were found among clade ages as taxa are increasingly undersampled. For the 85-taxon subsampling set where taxa were randomly deleted within the genus *Liolaemus*, the mean crown age of this clade was 29 and 33.6 MYA when only 3 and 5 taxa were sampled, respectively, compared to 47.6 for all 85 taxa (Table 1). Crown clade ages of deeper nodes in the tree such as Liolaemini also were affected and estimated to have diverged 83.7 MYA when only three *Liolaemus* taxa were randomly sampled compared to 111.3 MYA with the complete 85-taxon sampling. In fact, the sister taxon *Phymaturus*, whose sampling remained the same through all subsampling schemes, also had a reduced crown clade age of 28.9 MYA with three *Liolaemus* sampled compared to a crown clade age of 39.6 MYA (Table 1). The trend for all five focal clades was that increasing the number of taxa deleted from the tree had the effect of making the mean divergence times younger with very few exceptions.

For subsampling strategies two (randomly deleting taxa in only subgenus *Eulaemus*) and three (randomly deleting taxa in only subgenus *Liolaemus*), a considerable difference in crown ages of these groups was recovered with extensive undersampling. When only three and five taxa are sampled from the full 49-taxon clade *Eulaemus*, the estimated crown age of this group is reduced from 35.8 MYA to 19.3 and 21.5, respectively (Table 2). As above, the subgenus *Liolaemus* in subsampling set two also was affected by subsampling its sister clade showing a reduced crown clade age of 29.4 MYA compared to 39 MYA with a fully sampled dataset. In the final subsampling datasets when the taxa in the subgenus *Liolaemus* are undersampled with only three and five taxa, the crown ages of this clade are 25.3 and 28 MYA, respectively compared to 39 MYA with complete sampling of 36 taxa (Table 3). Again there is an effect on the age estimates of the sister clade (subgenus *Eulaemus*) with crown ages becoming younger with 35.8 MYA for complete sampling of subgenus *Liolaemus* taxa to 25.8 MYA when only 3 taxa were sampled.

### 3.4. Effect of One or Two Calibrations on Divergence Time Estimates

When only the node representing the common ancestor of Iguania was used as the calibration point in the complete data set, all divergence time estimates of crown clade ages across the tree were younger (Tables 4 and 5). At the deeper level of the tree, the crown clade age of Liolaemini was younger by between 12 and 16 million years. The genera *Phymaturus* and *Liolaemus* had crown clade ages that were younger by six and seven million years, respectively. The subgenera *Eulaemus* and *Liolaemus* each had clade ages that were 30.4 and 33.1 compared to 35.8 and 39.0 MYA when the full complement of eleven calibrations was used (Table 4). When all these datasets were rerun including the former single, deep calibration and a shallow, phylogenetically closer calibration fossil (*Pristidactylus*), results were very similar to one-calibration analyses with most node ages increasing in age by approximately 1–3 million years (Table 5). 

I examined the combined effect of using fewer calibrations when taxa were undersampled using subsampling set 1 (randomly deleting among 85 *Liolaemus* taxa). This strategy is similar to the sampling used by Fontanella et al. [10] except that there were no taxa sampled outside Liolaemini in that study. When at least 70 taxa are randomly deleted from the full complement of ingroup *Liolaemus* taxa, the remaining 15 taxa in *Liolaemus* yield mean crown clade estimates of 29.6 MYA using one calibration point and 29.5 MYA for two calibrations. This is 18 million years younger than the mean age of 47.6 MYA using all calibrations and full sampling and translates to the initial diversification event in the genus *Liolaemus* in the Middle Eocene to Early Oligocene. The mean crown clade ages of the two subgenera also are substantially lower when only 15 taxa are sampled. Using one or two calibrations gives similar estimates for the crown ages of subgenera *Eulaemus* around 19 MYA and *Liolaemus* at 23.5 MYA, respectively, compared to 35.8 and 39 MYA using all calibrations. The age estimate obtained by Fontanella et al. [10] for the crown age of *Eulaemus* sampling 17 taxa and using one calibration point was 18.08 MYA, which is very similar to the estimate of 19 MYA here also using a single calibration, and 15 sampled taxa. These differences again move initial diversification of these groups from the Late Eocene to near the boundary of the Early Miocene and Late Oligocene. 

For Bayesian analyses, when using only the single calibration of the common ancestor of Iguania, all ingroup node age estimates were older for both the IGR-exponential and uniform models compared to using all eleven calibrations. Differences between these estimates ranged from 14 to 35 million years across crown clades. In contrast to the eleven calibration analysis using complete taxon sampling, the IGR uniform model estimated all crown clade ages to be slightly older compared to estimates from the IGR exponential model. When the ancestral iguanian node calibration was combined with the more recent calibration of the node representing the *Pristidactylus* fossil, all age estimates were intermediate between the age estimates from the single calibration and complete eleven calibration estimates (Table 6).

## 4. Discussion

Penalized likelihood node age estimates reported here using undersampled clades and either one or two calibration fossils yield divergence times much younger compared to clades densely sampled and using eleven internal calibrations. The range of differences in mean clade ages within Liolaemini spanned from more than 45% younger when subgenus *Eulaemus* was significantly undersampled (sampling only 3 out of 49 taxa) while most other ages were between 20 and 30% younger using other subsampling schemes. It should be noted that node age estimates from this study should still be considered as minimum estimates as less than half the ~270 species within Liolaemini were sampled as well as less than one quarter of all species in Iguanidae. I expect all node ages estimated here to increase with increased taxon sampling.

Several studies have directly compared the results of divergence time estimates from PL and Bayesian methods [4, 39, 40]. For two of these studies [39, 40], Bayesian age estimates for focal nodes consistently were younger than PL divergence time estimates. However, Linder et al. [4] found that when taxa were densely sampled, Bayesian node age estimates were always older than PL estimates although undersampling different numbers of taxa in this latter study did not yield a consistent pattern for either method. The results of the present work show Bayesian node age estimates to be older than PL estimates when taxa are densely sampled and either all, one, or two calibration points are used.

Penalized likelihood is generally robust to model violations and taxon undersampling [4, 7, 25, 26], but a clear pattern can be seen in all subsampled datasets randomly removing taxa from individual clades. The more taxa removed from a particular clade, the younger age estimates of the nodes subtending those clades. This result is shown to affect node ages throughout the tree, clades directly undersampled, groups closely related to the focal clade, and node ages of clades deep in the tree. For example, the crown clade age of Iguanidae was estimated to be up to 15 million years younger by undersampling *Liolaemus* taxa (results not shown). Linder et al. [4] showed no effect on divergence time estimates from undersampling individual clades but admit that this aspect was not rigorously tested since most clades were represented by less than 30% of extant species.

Recently, advances in sequencing technologies have facilitated acquisition of dozens to thousands of nuclear loci for a few taxa with many of these studies publishing timetrees. For example, over the last decade, there have been numerous multigene nuclear and mtDNA studies published estimating divergence times within squamate reptiles [40–43]. For some of these studies, divergence times among clades sampled here are generally similar [41] but other studies attempting to estimate the age of Iguanidae, and Liolaemini along with its contained clades drastically undersample taxa using, in some cases, less 1% of extant species in a particular clade. As shown here and elsewhere [4], such extreme taxon undersampling will yield younger divergence time estimates regardless of dating methodology or data types used.

Numerous studies have attempted to estimate the node ages in the South American lizard clade Liolaemini [8–10, 44–47]. With the exception of the former two studies, these studies have focused on estimating divergence times within species complexes and closely related groups shallow in *Liolaemus* phylogeny using either a single calibration point or molecular evolutionary rate estimates. Most divergence times among lineages in these latter studies were recovered as occurring in the Pliocene and Pleistocene and are likely to be underestimates. This led the authors of those works to conclude that diversification among species groups within *Liolaemus* was most likely driven by glacial cycling processes over the last 3.5 million years. I agree that glacial cycling in the higher elevation regions of the Andes and the southern tip of South America likely played a significant role in shaping current diversity within *Liolaemus,* but as shown here, most divergence events among species occurred prior to the onset of Pliocene-Pleistocene glaciations. So that the primary impact of the glacial cycling is likely to be in shaping the current distributions and phylogeographic breaks within species rather than between species in most cases.

## 5. Conclusions

It is recommended that future studies at any phylogenetic level utilize as many calibration points as possible to obtain accurate divergence times in all parts of the tree. In those cases where very few or no calibration points may be available for ingroup taxa sampled, this study has shown that it is better to include many closely related and distant outgroups where multiple calibrations can be applied to improve divergence time estimation. Otherwise, node ages should be considered minimum ages for future studies and caution should be taken when attempting to make inferences of evolutionary processes driving diversification of modern taxa.

## Supplementary Material

The supplemental material contains a table of all GenBank accession numbers for sequences used in the phylogenetic analysis of this study. Museum specimen voucher and locality information for each sequence are provided in the GenBank accession record.

## Figures and Tables

**Table 1:** Mean divergence time estimates in MYA for higher-level Liolaemini lizard clades for the complete data set, ten internal calibration fossils, and subsampling set 1. Clade names correspond to names in Figure 1. SD is standard deviation as calculated in r8s1.8.

No. taxa deleted	Clade name				
	Liolaemini	*Phymaturus *	*Liolaemus* (genus)	*Eulaemus* (subgenus)*	*Liolaemus* (subgenus)*
None	111.3 (SD = 10.8)	39.6 (SD = 5.4)	47.6 (SD = 5.9)	35.8 (SD = 4.8)	39.0 (SD = 5.6)
3	90.1 (SD = 10.3)	31.9 (SD = 3.7)	36.9 (SD = 4.2)	28.0 (SD = 3.2)	30.1 (SD = 3.4)
5	89.0 (SD = 10.5)	31.6 (SD = 3.7)	36.5 (SD = 4.2)	27.7 (SD = 3.2)	29.7 (SD = 3.5)
15	89.4 (SD = 10.4)	31.7 (SD = 3.7)	36.7 (SD = 4.3)	27.6 (SD = 3.3)	29.8 (SD = 3.6)
30	86.8 (SD = 8.9)	30.7 (SD = 3.2)	35.5 (SD = 3.7)	26.7 (SD = 2.7)	28.7 (SD = 3.2)
50	85.2 (SD = 9.3)	30.1 (SD = 3.3)	34.7 (SD = 3.8)	24.9 (SD = 3.1)	27.7 (SD = 2.7)
70	86.6 (SD = 8.3)	30.4 (SD = 2.9)	35.3 (SD = 3.3)	23.2 (SD = 3.9)	28.2 (SD = 3.2)
80	84.7 (SD = 8.3)	29.5 (SD = 3.0)	33.6 (SD = 3.0)	N/A	N/A
82	83.7 (SD = 7.4)	28.9 (SD = 2.7)	29.0 (SD = 6.5)	N/A	N/A

*Divergence times for subgenera *Eulaemus* and *Liolaemus* in subsampling sets 80 and 82 taxa deleted were not calculated because in some resampling data sets, less than two taxa were sampled from that particular clade and a divergence time could not be calculated.

**Table 2:** Mean divergence time estimates in MYA for higher-level Liolaemini lizard clades for subsampling set 2 (deletion of taxa from subgenus *Eulaemus*) with all ten internal calibration fossils. Clade names correspond to names in Figure 1. SD is standard deviation as calculated in r8s1.8.

No. taxa deleted	Clade name				
	Liolaemini	*Phymaturus *	*Liolaemus* (genus)	*Eulaemus* (subgenus)	*Liolaemus* (subgenus)
None	111.3 (SD = 10.8)	39.6 (SD = 5.4)	47.6 (SD = 5.9)	35.8 (SD = 4.8)	39.0 (SD = 5.6)
3	86.9 (SD = 9.4)	30.8 (SD = 3.3)	35.6 (SD = 3.8)	27.0 (SD = 2.9)	29.1 (SD = 3.1)
5	87.0 (SD = 8.6)	30.8 (SD = 3.0)	35.6 (SD = 3.5)	27.0 (SD = 2.7)	29.1 (SD = 2.9)
15	87.7 (SD = 9.0)	31.0 (SD = 3.2)	35.8 (SD = 3.8)	27.0 (SD = 2.9)	29.3 (SD = 3.0)
30	84.2 (SD = 7.3)	29.5 (SD = 2.5)	34.4 (SD = 3.0)	24.9 (SD = 2.8)	28.2 (SD = 2.5)
44	83.7 (SD = 6.5)	29.0 (SD = 2.2)	34.3 (SD = 2.8)	21.5 (SD = 3.7)	28.4 (SD = 2.3)
46	86.6 (SD = 9.7)	30.0 (SD = 3.4)	35.7 (SD = 4.2)	19.3 (SD = 4.1)	29.4 (SD = 3.4)

**Table 3:** Mean divergence time estimates in MYA for higher-level Liolaemini lizard clades for subsampling set 3 (deletion of taxa from subgenus *Liolaemus*) with all ten internal calibration fossils. Clade names correspond to names in Figure 1. SD is standard deviation as calculated in r8s1.8.

No. taxa deleted	Clade name				
	Liolaemini	*Phymaturus *	*Liolaemus* (genus)	*Eulaemus* (subgenus)	*Liolaemus* (subgenus)
None	111.3 (SD = 10.8)	39.6 (SD = 5.4)	47.6 (SD = 5.9)	35.8 (SD = 4.8)	39.0 (SD = 5.6)
3	89.4 (SD = 11.1)	31.8 (SD = 4.0)	36.7 (SD = 4.6)	27.9 (SD = 3.5)	30.0 (SD = 3.7)
5	87.7 (SD = 10.4)	31.2 (SD = 3.7)	35.9 (SD = 4.4)	27.2 (SD = 3.2)	29.3 (SD = 3.6)
15	90.0 (SD = 10.9)	32.2 (SD = 3.9)	36.8 (SD = 4.5)	27.8 (SD = 3.4)	29.7 (SD = 3.6)
30	85.6 (SD = 8.3)	30.5 (SD = 3.0)	34.8 (SD = 3.5)	25.9 (SD = 2.6)	28.0 (SD = 2.7)
33	86.3 (SD = 9.1)	30.6 (SD = 3.2)	35.4 (SD = 4.0)	25.8 (SD = 3.0)	25.3 (SD = 6.4)

**Table 4:** Mean divergence time estimates in MYA for higher-level Liolaemini lizard clades using subsampling strategy 1 of Table 1 and only the root node calibration of Iguania with minimum and maximum ages of 144 and 218 MYA, respectively. Clade names correspond to names in Figure 1. SD is standard deviation as calculated in r8s1.8.

No. taxa deleted	Clade name				
	Liolaemini	*Phymaturus *	*Liolaemus* (genus)	*Eulaemus* (subgenus)*	*Liolaemus* (subgenus)*
None	94.5 (SD = 15.0)	33.5 (SD = 5.6)	40.4 (SD = 7.2)	30.4 (SD = 5.9)	33.1 (SD = 6.4)
3	77.9 (SD = 12.9)	27.6 (SD = 4.5)	31.9 (SD = 5.3)	24.2 (SD = 4.0)	26.0 (SD = 4.3)
5	74.1 (SD = 9.9)	26.3 (SD = 3.5)	30.4 (SD = 4.1)	23.1 (SD = 3.1)	24.8 (SD = 3.3)
15	80.3 (SD = 13.1)	28.5 (SD = 4.6)	33.0 (SD = 5.6)	24.8 (SD = 4.0)	26.8 (SD = 4.6)
30	77.6 (SD = 11.9)	27.4 (SD = 4.2)	31.7 (SD = 4.9)	23.9 (SD = 3.7)	25.7 (SD = 4.2)
50	71.4 (SD = 3.9)	25.2 (SD = 1.3)	29.0 (SD = 1.7)	20.9 (SD = 1.7)	23.4 (SD = 1.6)
70	72.6 (SD = 4.8)	25.4 (SD = 1.7)	29.6 (SD = 2.2)	19.3 (SD = 2.4)	23.6 (SD = 1.6)
80	73.2 (SD = 6.2)	25.5 (SD = 2.1)	29.0 (SD = 2.3)	N/A	N/A
82	72.0 (SD = 4.3)	24.9 (SD = 1.4)	24.8 (SD = 4.9)	N/A	N/A

*Divergence times for subgenera *Eulaemus* and *Liolaemus* in subsampling sets 80 and 82 taxa deleted were not calculated because in some resampling data sets, less than two taxa were sampled from that particular clade and a divergence time could not be calculated.

**Table 5:** Mean divergence time estimates in MYA for higher-level Liolaemini lizard clades using subsampling strategy 1 of Table 1 and the root node calibration of Iguania and the *Pristidactylus* fossil calibration ([38], Table 7). Clade names correspond to names in Figure 1. SD is standard deviation as calculated in r8s1.8.

No. taxa deleted	Clade name				
	Liolaemini	*Phymaturus *	*Liolaemus* (genus)	*Eulaemus* (subgenus)*	*Liolaemus* (subgenus)*
None	97.6 (SD = 16.4)	34.7 (SD = 6.7)	41.7 (SD = 7.7)	31.3 (SD = 6.0)	34.1 (SD = 6.6)
3	76.6 (SD = 11.4)	27.2 (SD = 4.1)	31.4 (SD = 4.7)	23.9 (SD = 3.5)	25.6 (SD = 3.8)
5	76.5 (SD = 9.9)	27.1 (SD = 3.6)	31.4 (SD = 4.1)	23.8 (SD = 3.1)	25.6 (SD = 3.3)
15	74.4 (SD = 8.5)	26.4 (SD = 3.0)	30.5 (SD = 3.6)	23.0 (SD = 2.7)	24.8 (SD = 3.1)
30	75.3 (SD = 11.4)	26.6 (SD = 4.0)	30.8 (SD = 4.8)	23.2 (SD = 3.6)	24.9 (SD = 3.9)
50	75.2 (SD = 8.0)	26.6 (SD = 2.8)	30.6 (SD = 3.5)	22.0 (SD = 2.4)	24.7 (SD = 2.8)
70	72.2 (SD = 3.6)	25.3 (SD = 1.4)	29.5 (SD = 1.7)	19.2 (SD = 2.3)	23.5 (SD = 1.6)
80	71.3 (SD = 3.6)	24.8 (SD = 1.4)	28.3 (SD = 1.6)	N/A	N/A
82	74.4 (SD = 9.7)	25.7 (SD = 3.3)	25.9 (SD = 6.8)	N/A	N/A

*Divergence times for subgenera *Eulaemus* and *Liolaemus* in subsampling sets 80 and 82 taxa deleted were not calculated because in some resampling data sets, less than two taxa were sampled from that particular clade and a divergence time could not be calculated.

**Table 6:** Comparison of node age estimates obtained using three fossil calibration sampling schemes in MrBayes. Values represent mean and 95% posterior credibility intervals (in parentheses) for the age of each node. All analyses implemented the independent gamma rates (IGR) relaxed clock model, a continuous uncorrelated model. This model explained the data better than the other two relaxed clock models currently implemented in MrBayes. The prior parameter specifying the rate of increase over time in variance of effective branch lengths was drawn from either a uniform or exponential distribution as either explained the data equally well according to Bayes Factors calculated in Tracer. Clade names correspond to names in Figure 1.

Assumed model	Clade name				
	Liolaemini	*Phymaturus *	*Liolaemus* (genus)	*Eulaemus* (subgenus)	*Liolaemus* (subgenus)
IGR-exponential All calibrations	121.0 (102–143)	42.1 (31–55)	60.3 (50–71)	38.0 (29–46)	53.4 (44–64)
IGR-exponential Iguania ancestor only	143.4 (121–177)	56.8 (36–77)	92.3 (76–113)	73.5 (58–91)	79.7 (64–99)
IGR-exponential Iguania ancestor and *Pristidactylus *	132.9 (96–170)	50.9 (30–77)	81.9 (53–108)	63.2 (42–84)	70.7 (47–94)
IGR-uniform All calibrations	114.7 (87–148)	39.8 (26–53)	57.3 (43–72)	36.2 (27–45)	50.5 (38–64)
IGR-uniform Iguania ancestor only	147.5 (123–174)	57.0 (36–77)	93.2 (73–116)	73.9 (54–95)	80.9 (62–102)
IGR-uniform Iguania ancestor and *Pristidactylus *	N/A*	53.2 (34–72)	86.5 (63–112)	68.2 (49–90)	74.6 (54–99)

*Divergence times for Liolaemini were not included because the clade credibility tree did not recover this clade as monophyletic.

**Table 7:** Squamate fossils and dates in millions of years (mya) used as calibration points.

Date (MYA)		Fossil taxon	Node assigned age	Reference
	218–144	Stem age of Iguania (maximum–minimum)	This age range was chosen to include the estimated divergence dates from several recent analyses of squamate and iguanian relationships. See arguments made by authors in associated references	[41, 48–51]
(1)	33.5	*Crotaphytusoligocenicus *	Stratigraphic age corresponding to the beginning of lower Oligocene placed at common ancestor of *Crotaphytus* and *Gambelia *	([52] (but see [53]))
(2)	20	*Dipsosaurus sp. *	Stratigraphic age corresponding to approximate midpoint (16–23) of lower Miocene placed at common ancestor of *Dipsosaurus* and its sister group	[53]
(3)	2.7	*Sceloporus undulatus* clade	Approximate midpoint stratigraphic age of Pliocene (1.8–3.6) placed at common ancestor of taxa referred to as members of *Sceloporus undulatus* clade and their sister group	[53]
(4)	14.5	*Sceloporus *	Approximate midpoint of stratigraphic age of fossil horizon (12–17 mya) placed at common ancestor of *Sceloporus* and its sister group	[53, 54]
(5)	31	*Paraphrynosoma greeni*	Midpoint stratigraphic age (28–33 mya) of fossil horizon placed at common ancestor of *Phrynosoma* and the sand lizard clade	[55]
(6)	13.5	*Holbrookia antigua *	Midpoint stratigraphic age (11–16 mya) of fossil horizon placed at common ancestor of *Holbrookia* and *Cophosaurus *	[56]
(7)	50.3	*Suzanniwana patriciana *	Youngest stratigraphic age of Wasatchian time period (55.8–50.3 MYA) placed at the common ancestor representing crown Corytophanidae	[57]
(8)	16	*Liolaemus sp. *	Approximate age of the end of the Early Eocene from with the fossil was collected placed at crown of genus *Liolaemus *	[36, 38]
(9)	16	*Pristidactylus sp. *	Approximate age of the end of the Early Eocene from with the fossil was collected placed at the common ancestor of *Pristidactylus* and its sister taxon	[36, 38]
(10)	33.9	*Polychrus charisticus *	Midpoint stratigraphic age (30.5–36.5 mya) of fossil horizon placed at common ancestor of *Polychrus* and its sister group	[58]

## Appendix

See Table 7.
