# Supplementary material for: Undersampling Taxa Will Underestimate Molecular Divergence Dates: An Example from the South American Lizard Clade Liolaemini
Source: Int J Evol Biol. 2013 Oct 9;2013:628467. doi: 10.1155/2013/628467 (PMC3809987; doi:10.1155/2013/628467)
Supplement: Supplementary file 1 — The supplemental material contains a table of all GenBank accession numbers for sequences used in the phylogenetic analysis of this study. Museum specimen voucher and locality information for each sequence are provided in the GenBank accession record. [file 628467.f1.pdf]

Supplemental material for "Undersampling taxa will underestimate molecular divergence dates: An example from the South American lizard clade Liolaemini " by James A. Schulte II

Table S1. GenBank accession numbers for DNA sequences used for phylogenetic analysis in this study.

TAXON	ACCESSION NO.
Elgaria panamitina	U82692
Varanus griseus	AF407503
Cnemidophorus tigris	U71332
Mabuya aurata	U71330
Brookesia perarmata	AF448776
Chamaeleo dilepis	AF128460
Leiolepis belliana	U82689
Anisolepis longicauda	AF528736
Anolis cristatellus	AF528724
Anolis cybotes	AF528723
Anolis distichus	AF528725
Anolis paternus	U82679
Anolis punctatus	AF528726
Anolis sagrei	AF528727
Basiliscus galeritus	AF528714
Basiliscus plumifrons	U82680
Basiliscus vittatus	AF528715
Brachylophus fasciatus	AF528721
Callisaurus draconoides	AY297492
Chalarodon madagascarensis	AF528722
Cophosaurus texana	AY297489
Corytophanes cristatus	AF528717
Corytophanes percarinatus	AF528718
Crotaphytus collaris	U82681
Ctenosaura palearis162073DONE	GQ502763
Ctenoblepharys adspersa	AF305784
Diplolaemus altopatagonica	AF528728
Diplolaemus darwinii	AF528729
Dipsosaurus dorsalis	AF049857
Enyalioides laticeps	AF528719
Enyalius leechii	AF528733

Gambelia copei	GQ502765
Gambelia wislizenii	U82682
Holbrookia maculata	AY297490
Holbrookia propinqua	AY297491
Hoplocercus spinosus	U82683
Laemanctus longipes	AF528716
Leiocephalus carinatus	AF049864
Leiocephalus personatus	AF528754
Leiocephalus schreibersi	AF528753
Leiosaurus bellii	AF528730
Leiosaurus catamarcensis	AF528731
Liolaemus abaucan	AF099263
Liolaemus albiceps	AF099267
Liolaemus andinus	AF099245
Liolaemus andinus2	AF305789
Liolaemus audituvelatus	AF305792
Liolaemus austromendocinus	AF099239
Liolaemus bellii	AF099223
Liolaemus bibronii	AF099221
Liolaemus bitaeniatus	AF099219
Liolaemus darwinii (old boulengeri)	AF099275
Liolaemus buergeri	AF099236
Liolaemus canqueli	AY297536
Liolaemus capillitas	AF099234
Liolaemus ceii	AF099237
Liolaemus cf. poecilochromus	DQ002485
Liolaemus rothi (oldmelanops)	AF099261
Liolaemus chacoensis	AF099270
Liolaemus chaltin	AF099218
Liolaemus chiliensis	AF099224
Liolaemus chlorostictus	AF099247
Liolaemus coeruleus	AF099217
Liolaemus curis	GQ502779
Liolaemus cuyanus	AF099252
Liolaemus cuyanus FBC 130	DQ002486
Liolaemus cuyanus FBC 67	DQ002487
Liolaemus darwiniiLaRioja	AF099274
Liolaemus cf. darwinii FBC146	DQ002490
Liolaemus scrocchi	AF099248
Liolaemus elongatus	AF099240

Liolaemus fabiani	AF305793
Liolaemus famatinae	AF099246
Liolaemus fitzingerii	AF099253
Liolaemus fuscus	AF099232
Liolaemus gracilis	AF099222
Liolaemus gravenhorsti	AY297527
Liolaemus grosseorum	AF099272
Liolaemus huacahuasicus	AY297533
Liolaemus incaicus alticolor	GQ502778
Liolaemus irregularis	AF099268
Liolaemus isabelae	GQ502776
Liolaemus josephorum	GQ502777
Liolaemus kingii	GQ502782
Liolaemus koslowskyi	AF099264
Liolaemus kriegi	AY297530
Liolaemus laurenti	AF099273
Liolaemus lemniscatus	AF099229
Liolaemus leopardinus	AF099235
Liolaemus lineomaculatus	AF099241
Liolaemus lutzae	AF099255
Liolaemus magellanicus	AF099243
Liolaemus melanops FBC45	DQ002489
Liolaemus melanops FBC58	DQ002488
Liolaemus molinai	AF305915
Liolaemus monticola	AF099230
Liolaemus multicolor	AF099250
Liolaemus multimaculatus	AF099257
Liolaemus nigroviridis	AF099233
Liolaemus nigroviridis campanae	GQ502774
Liolaemus nitidus	AF099231
Liolaemus occipitalis	AF099256
Liolaemus olongasta	AF099271
Liolaemus ornatus	AF099266
Liolaemus paulinae	AY297531
Liolaemus petrophilus	AF099238
Liolaemus pictus (old cyanogaster)	AF099225
Liolaemus pictus (old cf. hernani)	AY297527
Liolaemus pictus Rio Negro	U82684
Liolaemus pictus neuquen	AF099226
Liolaemus platei	AY297528

Liolaemus poecilochromis	AF099249
Liolaemus polychromus	GQ502780
Liolaemus pseudoanomalus	AF099254
Liolaemus quilmes	AF099265
Liolaemus robertmertensi	AF099220
Liolaemus rothi	AF099262
Liolaemus ruibali	AF099244
Liolaemus salinicola	AF099259
Liolaemus scapularis	AF099258
Liolaemus somuncurae	AF099242
Liolaemus sp nigromaculatus	AY297526
Liolaemus telseni FBC62	DQ002491
Liolaemus tenuis	AF099228
Liolaemus tenuis punctatissimus	GQ502775
Liolaemus torresi	GQ502781
Liolaemus uspallatensis	AF099269
Liolaemus wiegmannii	AF099260
Liolaemus zapallarensis	AF099227
Microlophus atacamensis	AF528752
Microlophus koepckeorum	GQ502766
Morunasaurus annularis	AF528720
Oplurus cuvieri	U82685
Petrosaurus thalassinus	AF049858
Petrosaurus.mearnsi	L40444; L41450
Phrynosoma cornutum	AY297487
Phrynosoma coronatum	AY297485
Phrynosoma hernandesi	U82686
Phrynosoma mcallii	AY297486
Phrynosoma modestum	AY297484
Phrynosoma platyrhinos	AY297488
Phrynosoma solare	AF528739
Phrynosoma asio	GQ502772
Phrynosoma ditmarsi	GQ502771
Phrynosoma orbicularae	GQ502770
Phrynosoma taurus	GQ502769
Phymaturus antofogasta	AY661892
Phymaturus indistinctus	AY661893
Phymaturus palluma	AF099216
Phymaturus patagonicus	AY661894
Phymaturus somuncurensis	AF049865

Phymaturus zapalensis	GQ502773
Polychrus acutirostris	AF528737
Polychrus marmoratus	AF528738
Pristidactylus scapulatus	AF528732
Sator angustus	AF049859
Sauromalus obesus	U82687
Sceloporus adleri	AY297519
Sceloporus carinatus	AY297496
Sceloporus cautus	AY297522
Sceloporus clarki	AY297511
Sceloporus cyanogenys	AY297524
Sceloporus formosus	AY297498
Sceloporus graciosus	AF049860
Sceloporus grammicus	AY297509
Sceloporus hunsakeri	AY297506
Sceloporus jalapae	AY297504
Sceloporus jarrovii	AY297512
Sceloporus lundelli	AY297499
Sceloporus maculosus	AY297501
Sceloporus magister	AF528741
Sceloporus malachiticus	AY297518
Sceloporus merriami	AY297520
Sceloporus mucronatus	AY297497
Sceloporus occidentalis	AY297515
Sceloporus ochoterenae	AF528743
Sceloporus olivaceous	AY297521
Sceloporus orcutti	AY297508
Sceloporus ornatus	AY297523
Sceloporus pictus	AY297500
Sceloporus poinsettii	AY297510
Sceloporus pyrocephalus	AY297502
Sceloporus scalaris	AF528742
Sceloporus siniferus	AY297494
Sceloporus smaragdinus	AY297517
Sceloporus spinosus	AY297525
Sceloporus squamosus	AY297495
Sceloporus teapensis	AY297505
Sceloporus undulatus NewMex	AY297514
Sceloporus utiformis	AF528740
Sceloporus variabilis	AY297507

Sceloporus virgatus	AY297516
Sceloporus woodi	AY297513
Sceloporus zosteromus	AY297503
Stenocercus crassicaudatus	AF049866
Stenocercus doellojuradoi	AF528744
Stenocercus empetrus	DQ080219
Stenocercus ochoai	AF528746
Tropidurus etheridgei	AF528750
Tropidurus plica	AF528748
Tropidurus spinulosus	AF528751
Uma scoparia	AF049861
Uracentron flaviceps	AF528747
Uranoscodon superciliosa	AF528749
Urosaurus graciosus	AF049862
Urosaurus ornatus	AY297493
Urostrophus gallardoi	AF528735
Urostrophus vautieri	AF528734
Uta stansburiana	AF049863
